# Classification Models for Skin Tumor Detection Using Texture Analysis in Medical Images

**DOI:** 10.3390/jimaging6060051

**Published:** 2020-06-19

**Authors:** Marcos A. M. Almeida, Iury A. X. Santos

**Affiliations:** 1 Departamento de Eletrônica e Sistemas, Centro de Tecnologia, Universidade Federal de Pernambuco, Recife-PE 50670-901, Brazil; 2 Departamento de Física, Universidade Federal Rural de Pernambuco, Recife-PE 52171-900, Brazil; iuryadones@gmail.com

**Keywords:** texture analysis, melanoma, glcm matrix, machine learning, classifiers

## Abstract

Medical images have made a great contribution to early diagnosis. In this study, a new strategy is presented for analyzing medical images of skin with melanoma and nevus to model, classify and identify lesions on the skin. Machine learning applied to the data generated by first and second order statistics features, Gray Level Co-occurrence Matrix (GLCM), keypoints and color channel information—Red, Green, Blue and grayscale images of the skin were used to characterize decisive information for the classification of the images. This work proposes a strategy for the analysis of skin images, aiming to choose the best mathematical classifier model, for the identification of melanoma, with the objective of assisting the dermatologist in the identification of melanomas, especially towards an early diagnosis.

## 1. Introduction

The skin cancer is among the most common types of cancer in the world [1]. Melanoma is the most dangerous type of skin cancer, caused by over production of melanin pigments that change the color and texture of skin, resulting as a dark area on the skin [2]. Data indicate that the incidence of melanoma, which is a type of cancer that metastasizes rapidly, has increased alarmingly [3].

However, visual analysis is limited by human visual ability, as well as human perception and sensitivity, in addition to the fact that not all melanomas have the same characteristics. The tumor is an exceptional expansion of human cells that reproduce in an unrestricted way and that can be identified by a variation of color and texture of the human tissue under study, making information contained in the images extremely valuable. Textures are visual patterns, which have brightness, color, slope, size and other attributes. When partitioned into sub-images by regions of interest, they are able to be properly classified.

**Figure 1:**
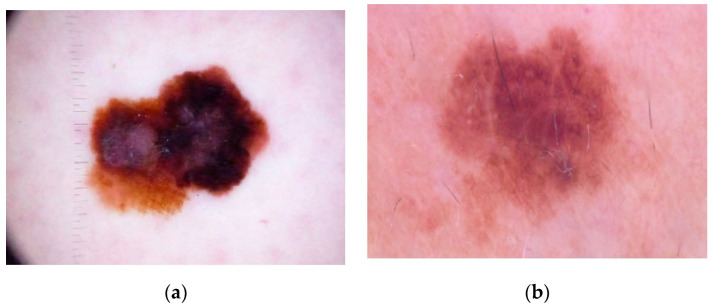
Images of melanoma and nevus tissues: (**a**) Skin lesion from melanoma image; (**b**) Nevus Skin.

Color is one of the significant features in the examination of skin lesion. The distribution of texture and color features presents significant information, as Figure 1 shows.

A technique of analysis that focuses on the extraction of intrinsic characteristics of the image such as—brightness and color, providing an idea of the roughness or smoothness, among other characteristics, is texture analysis. Digital image texture analysis refers to techniques that use image processing in order to extract representative features of the images studied which can have importance in the discrimination between images.

This makes it possible to accelerate decisions concerning diagnosis. In these cases, image quality is essential, relying on bandwidth, sensitivity, resolution and signal-to-noise ratio of the image systems. Artificial intelligence can be very useful in assisting oncologists and radiologists in making early diagnoses of tissue regions with melanoma [4].

**Figure 2:**
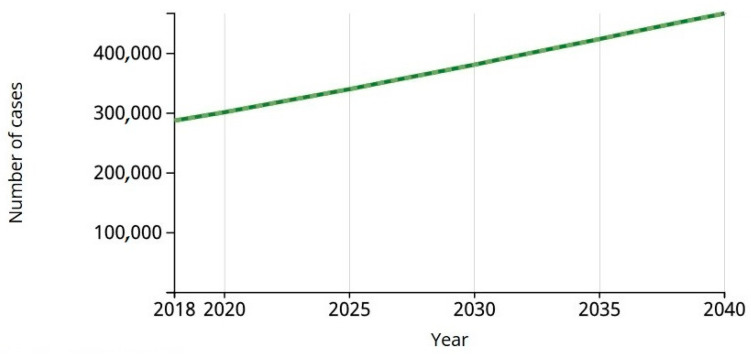
Estimated of incident cases from 2018 to 2040, melanoma of skin, both sexes, all ages. Source: International Agency for Research on Cancer of World Health Organization.

In Figure 2 a graphical representation of nearly 300,000 cases predicted indicates melanoma was the 19th most-incident tumor in the world in 2018 and will continue rising, according to the World Health Organization’s (WHO) International Agency for Research on Cancer (IARC) study Globocan [5].

The estimate foresees that, this year, 2020, 1.81 million new cases of cancer and 9.5 million deaths due to neoplasia will occur. One in five men and one in six women are predicted to develop the disease over a lifetime. This is in contrast to the last survey published, in 2012, when the International Agency for Research on Cancer (IARC of WHO) predicted 14.1 million new cases and 8.2 million deaths.

According to the IARC, by 2020 there are expected to be 300,000 new cases of melanoma in the world.

In 2018, the number of melanoma cases was 287,723. The estimate for 2040 will be 466,914. Estimated number of deaths from 2018 to 2040 will be 42,208, melanoma of skin, both sexes, all ages.

Given the growth of demand for early diagnosis, a tool such as computer vision with a machine learning tool could help radiologists to produce relevant diagnoses more quickly and provide quantitative measures for regions suspected of having melanoma cancer. The works related to this research reveal this trend.

In this article, the new strategy is proposed through the addition of texture information, through the red, green and blue color channels, called here RGB components. In the process of acquiring the characteristics of the textures, using the Python syntax and the algorithms in the Python library to pinpoint the location of the region of interest (ROI) in the skin image, regardless of the image position.

Artificial intelligence when applied to the set of data representative of characteristic textures of the images, can assist oncologists and radiologists in identifying suspicious skin regions. Adequate experimental results have shown how this strategy can provide an accurate measure of quality that correspond to the subjective assessments by clinical experts [6].

The human eye, despite its perfection, is not able to capture certain details in an image or to distinguish small differences between certain micro-textures. Using Machine Learning, these differences can be measured, evaluated and compared to standard images and thus identify microtextural differences in medical examination images. Different parameters of texture reflect different properties within the image.

The purpose of resource extraction is to choose a data set representative of the original image measuring certain properties or resources that can distinguish a pattern between one sub-image and another.

This is a problem of binary classification, since the samples belonged to two classes—nevus tissue or melanoma, so the proposal for a solution for this new strategy for detecting melanoma is presented. All experimental simulations were implemented and executed using the Python language.

The paper is organized as follows—Section 2 presents the studies related to research, introducing the most common techniques used for the detection of tumors.

Section 3, deals with the theoretical framework in first and second order statistics.

Section 4 proposes strategies and solutions using a machine learning application for the detection of skin cancer.

Section 5 is dedicated to presenting the results of the experiments. Some discussions are presented in Section 6 and in Section 7 we make conclusions from the results of this study, with suggestions for new lines of research and future work.

## 2. Related Works

Some research works are related to our study because they use convergent techniques for texture analysis.

The diagnosis of skin lesions was studied by Zhang [7]. The analysis considered Convolutional Neural Networks (CNN) for automatic detection of skin cancer, comparing this with other research methods. The proposed method called CNN/WOA achieved an accuracy of 91.00%, with a sensitivity of 95.00% and specificity of 91.00%.

Pathan [8] reviewed the cutting-edge techniques declared in the literature, summarizing these state of art approaches. The steps included dermoscopic image pre-processing, segmentation, extraction and selection of peculiar characteristics and disposition of the skin lesions. The study also evaluated the consequences of the methodologies reported in the literature in addition to the results and future directions of research. The best result from the listed methods and algorithms was the Otsu threshold with Active Contour using a Sparse-Field level-set method, with a precision ability of 97.50% for the detection of melanomas.

Lee et al. [9] proposed the skin disease classification solution using Fine-tuned Neural Networks. The model achieved an accuracy of 89.90% and 78.50% in the validation set and the test set, respectively.

Using the technique of aggregating robust convolutional neural networks (CNNs) into a structure, Harangi [10] achieved classification results in three classes of injuries. The experimental results concluded that the average area under the receiver operating characteristic curve (AUC) was 89.10% for the task of categorizing the 3 classes.

Li [11] proposed two deep learning methods to address three main tasks emerging in the area of skin lesion image processing, that is, lesion segmentation (task 1), lesion dermoscopic feature extraction (task 2) and lesion classification (task 3). The proposed deep learning frameworks were evaluated on the ISIC 2017 dataset. Experimental results show the promising accuracies of these frameworks, that is, 75.30% for task 1, 84.80% for task 2 and 91.20% for task 3 were achieved.

A method proposed by Abbadi [12], takes into account the techniques known as ABCD—Asymmetry, Edge, Color and Diameter. For the detection of melanoma, the metric TDS (Total Dermoscopy Score) was calculated to perform the classification. The accuracy found in the results was 95.45%.

Fernandez [3] proposed in his research the extraction of characteristics that appear in the image of the lesion and treated with the Gray Level Co-occurrence Matrix (GLCM) method. Then, during the detection phase, a set of classifiers determined the occurrence of a malignant tumor. The experiments were carried out on images obtained from the ISIC repository. The proposed system provides skin cancer detection accuracy above 88.00%.

Ansari [13] proposed a skin cancer detection framework using SVM for early detection of skin cancer. The dermoscopic image of skin cancer was obtained and submitted to different pre-processing strategies using filtering images. The GLCM system was used to select specific highlights on the image which were then used to help establish the classifier. The classification determined whether the image was of a cancerous or non-cancerous tissue. The accuracy of the proposed structure is 95.00%.

The following are references from other authors with their respective works and applications, using similar techniques for extracting characteristics in medical images.

The diagnosis of breast cancer subtypes using image texture analysis was studied by Waugh [14]. The analysis considered the distribution of the pixel intensities in the magnetic resonance images. The entropy parameters of the GLCM matrix resulted in significant contributions to image classification, which can be useful in the treatment and monitoring of breast cancer therapeutics.

Recently, Vamvakas [15] proposed the solution of a challenge in the diagnosis of magnetic resonance images, using advanced techniques such as Diffusion Tensor Imaging—distinguishing ambiguous images in the appearance of Glioblastoma Multiforme and solitary metastasis, using 3D textural resources with GLCM.

Jennitta [16] using GLCM and Local Standard Descriptor parameters, applied to magnetic resonance images of the brain, showed a promising approach to medical diagnosis.

Hiba Asri [17], with the objective of diagnosing breast cancer, used machine learning techniques, such as—Support Vector Machine (SVM), Decision Tree, Naive Bayes and K closest neighbors, in the Wisconsin Breast Cancer database. The results proved that the SVM had the best accuracy—97.13%.

In a recent study on lung cancer, Yoon et al. [18], selected parameters for texture analysis in magnetic resonance images. Correlation between tumor area and size was calculated by linear regression. The injection of contrast material was used to check the MRI images and improvements were recorded in the selected texture parameters, in a time window between 120–180 s.

A predictive and two probabilistic model for detecting cancer in the human liver using computed tomography images was shown by Seal [19]. Haralick [20] calculated parameters in the GLCM of images of the liver with injury and without injury, as made available with several classification models such as—Logistic Regression (LR), Linear Discriminant Analysis (LDA) and a predictive model using Multilayer Perceptron (MLP), to estimate the likelihood of a patient having liver cancer or not. It was proved that logistic regression (96.67%) obtained the best accuracy when compared to LDA (95.00%) and MLP (94.40%).

Harshavardhan [21] used the SVM (Support Vector Machine) to classify data extracted from brain images to characterize benign or malignant tumors. To evaluate the performance of these resources, several texture methods were used, such as the histogram, Gray Level Co-occurrence Matrix (GLCM), gray level execution length matrix (GRLM), all analyzed separately. Performance results ranged from 82.97% to 92.83%.

Bahadure [22] demonstrated an efficient proposal to identify normal and abnormal tissues from MRI images of the brain. The experimental results identified one classified with an accuracy of 96.51%, specificity of 94.20% and sensitivity of 97.72%. Machine learning techniques were used, with data on texture, color, contrast and GLCM of the studied images.

Abdel-Nasser et al. [23] proposed a method that generates a set of compact representations of infrared breast images, with competitive results (AUC = 0.989), able to differentiate between normal and cancerous cases.

## 3. Materials and Methods

There follows a general description of the statistical parameters used in the proposal of this work, for the extraction of characteristics from the images. These characteristics make up the co-occurrence matrix (GLCM) and are part of the keypoints, applied to each image.

Let f (*x*, *y*) be a function of two discrete variables *x* and y, *x* = 0, 1, …, *N* − 1 and *y* = 0, 1, …, *M* − 1. The discrete function f (*x*, *y*) can assume values for *i* = 0, 1, …, L − 1, where L is the number of grayscale levels. The intensity-level histogram is a function showing (for each intensity level) the number of pixels in the whole image, which have this intensity:

(1) $$h\left(i\right)=\overset{N-1}{\underset{x=0}{\sum }}\overset{M-1}{\underset{y=0}{\sum }}\delta \left(f\left(x,y\right),j\right),$$

where δ(*i*, *j*) is the Kronecker delta function

(2) $$\delta \left(i,j\right)=\{\begin{array}{c} 1.\;\;i=j\\ 0.\;\;i\neq j \end{array}.$$

The probability of occurrence of each pixel in the image that will appear in the histogram is given by:

(3) $$p\left(i\right)=\frac{h\left(i\right)}{M},\;\;\;\;\;\;\;\;\;i=0,\;1,\;\ldots .,\;L-1.$$

These resources are determined automatically, constituting the first order statistics (Equations (4)–(7)) and used at keypoints. The average intensity of the grayscale is calculated by:

(4) $$\mu =\overset{L-1}{\underset{i=0}{\sum }}ip\left(i\right).$$

The variance shows the degree of variability around the average grayscale distribution:

(5) $$\sigma^{2}=\overset{L-1}{\underset{i=0}{\sum }}{\left(i-\mu \right)}^{2}p\left(i\right).$$

Skewness measures the asymmetry of the histogram:

(6) $$\mu_{3}=\sigma^{-3}\overset{L-1}{\underset{i=0}{\sum }}{\left(i-\mu \right)}^{3}p\left(i\right).$$

Kurtosis is a measure of whether the data are heavy-tailed or light-tailed relative to the normal distribution. The kurtosis is a measure of flatness of the histogram.

(7) $$\mu_{4}=\sigma^{-4}\overset{L-1}{\underset{i=0}{\sum }}{\left(i-\mu \right)}^{4}p\left(i\right)-3.$$

The second-order histogram is defined as that Gray-Level Co-occurrence Matrix, that is a square matrix is formed by elements that indicate the probability of occurrence for a pair of pixels with intensities that depend on the distance d and the angle *θ*. Equations (8) through (14) make up the second-order statistics set.

(8) $$p\left(i,j,d,\;\theta \right)=\{\left(\left(x_{1},y_{1}\right),\left(x_{2},y_{2}\right)\right):h\left(x_{1},y_{1}\right)=\;i\;,\;h\left(x_{2},y_{2}\right)=j\},$$

where

(9) $$\left(x_{2},\;y_{2}\right)=\left(x_{1\;},\;y_{1}\right)+\left(dcos\theta ,dsen\theta \right).$$

In this study, the distances considered were d = 1, 2, …, 5, with angles *θ* = 0°, 45°, 90° and 135°.

Energy derived from the second angular momentum measures the local uniformity of the shades of gray.

(10) $$E=\sum_{i}\sum_{j}{\left[p\left(i,j\right)\right]}^{2}.$$

Entropy measures the degree of clutter between the image pixels:

(11) $$H=-\underset{i}{\sum }\underset{j}{\sum }p\left(i,j\right)log_{2}\left[p\left(i,j\right)\right].$$

Correlation is a measure of how a pixel is associated with its neighbor across the image and assumes values ranging from ±1.

(12) $$\rho =\overset{L-1}{\underset{i=0}{\sum }}\overset{L-1}{\underset{j=0}{\sum }}p\left(i,j\right)\frac{\left(i-\mu_{X}\right)\left(j-\mu_{Y}\right)}{\sigma_{X}\sigma_{Y}}.$$

Contrast is a difference moment of the GLCM and measures the amount of local variations in an image:

(13) $$C=\overset{L-1}{\underset{i=0}{\sum }}\overset{L-1}{\underset{j=0}{\sum }}{\left(i-j\right)}^{2}p\left(i,j\right).$$

Haralick [20] proposed a set of scalar quantities for summarizing the information contained in a GLCM. Originally these comprised a total of 14 features namely, angular second moment, contrast, correlation, sum of variance, inverse difference moment, sum average, sum variance, sum entropy, entropy, difference variance, difference entropy, information measures of correlation and maximal correlation coefficient. In order to obtain texture features, the normalized GLCM was computed for each of four orientations (0°, 45°, 90° and 135°).

GLCM expresses the texture feature according to the calculation of the conditional probability of the pixel pair of the gray intensities, for the different spatial positions [24].

(14) $$p\left(i,j|d,\theta \right)=\frac{p\left(i,j,d,\;\theta \right)}{\sum_{i}\sum_{j}p(i,j|d,\theta )}.$$

The next step was to format a proposal, containing the extraction of characteristics from medical images to build a classification based on several state-of-the-art classifiers.

## 4. Proposed Strategy

The present study investigates the best strategy for aiding in the diagnosis of the presence or absence of melanoma through skin imaging. In the proposed strategy, what differentiates it from other methods of texture analysis is the inclusion of RGB components, adding texture information to the keypoints.

The developed strategy involves the following steps:

1. Random selection of a set of images with melanoma and nevus.
2. Generation of keypoints containing:
  - first order statistics information;
  - second order statistics parameters;
  - RGB component information.
3. Extraction of characteristics from all training images.
4. Classification phase with the modeling using the training database.
5. Application of the selected model to a test image database.
6. Result of the model applied to the test database.

In addition, the algorithm used to read the keypoints does not depend on the image position, when capturing information in the region of interest—ROI.

**Figure 3:**
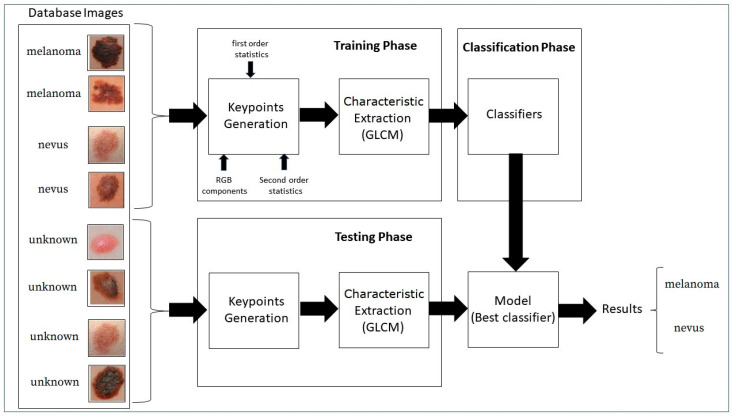
Block Diagram of the proposed strategy.

The block diagram in Figure 3 shows the proposed strategy.

All data sets used are available in the Skin Lesion Analysis Towards Melanoma—International Skin Imaging Collaboration (ISIC) 2019 [25].

The database was formed by 2000 jpeg skin images, selected at random. The learning process was performed on 75% of the database, as in this research, 10 samples were used per image, the learning process analyzed a total of 15,000 samples. In the testing process, the remaining 25% of the images in the database were used, making a total of 5000 samples in the testing phase. Each sample had a dimension of 6 pixels × 6 pixels.

To increase the efficiency in extracting characteristics for differentiation of the tissues, it was necessary to add parameters as first and second order statistics into keypoints, such as—mean, variance, kurtosis, skewness, contrast, correlation, entropy, energy, maximum and minimum value, as well as RGB components.

After extracting the characteristics of the images, modeling was performed through training the database, using the best-known classifiers and their variations, as found in the academic literature:

- Stochastic Gradient Classifier.
  The basic idea of this classifier method is straightforward—iteratively adjust the parameters *θ* in the direction where the gradient of the cost function is large and negative. In this way, the training procedure ensures the parameters flow towards a local minimum of the cost function.
- Naïve Bayes Classifier.
  A Naive Bayes classifier is a simple probabilistic classifier based on applying Bayes’ theorem (from Bayesian statistics) with strong (naive) independence assumptions. This classifier is among those common learning methods grouped by similarities which makes use of Bayes’ theorem of probability to build ML models, especially those related to disease prediction and document classification.
- Decision Tree Classifier.
  A decision tree is a decision support tool that uses a tree-like graph and its possible results. It is a way to display the algorithm.
- Random Forest Classifier.
  Random forests are an ensemble learning method for classification, regression and other tasks, that operate by constructing a multitude of decision trees at training time. As a result, the classes (classification) or average forecast (regression) of these individually generated trees are grouped.
  This method aims at averaging many approximately unbiased but noisy trees to obtain low variances results. Is a collection of decisions tress, which, together, forms a forest.
- KNN Classifier.
  Classification is achieved by identifying the nearest neighbors to a query example and using those neighbors to determine the class of the query.
- Support Vector Machine Classifier.
  The objective of the SVM classifier is to find the hyperplane that separates the points of classes *C*_1_ and *C*_2_ with a maximum margin, linearly penalizing points within the margin through a regularization parameter selected by the user.
  Support vector machines bring a new option to the pattern recognition problem with clear connections in statistical learning theory. They differ radically from other methods, for example, neural networks—the training of an SVM always finds a global minimum and its simple geometric interpretation provides much scope for deeper investigations.
- Model Logistic Regression Classifier.
  Logistic regression classifies by using the log-ratios between the probability of groups given the data. For the groups $g_{1}$ and $g_{2}$:
  (15) $$log\frac{P(G=g_{1}|X=x)}{P(G=g_{2}|X=x)}=\beta_{0}+x\;\beta_{x}=0.$$
  The decision boundary is the value where the probability of the group given the data is equal.
  To find it, the likelihood function of *β* is maximized:
  (16) $$L\left(\beta \right)=\overset{N}{\underset{i=1}{\sum }}logPg_{i}\left(x|\beta \right).$$

In machine learning, the classification identifies to which Class a set of observed data belongs. Classification is an example of pattern recognition. Some variants of the classifiers cited and available in the library of the Python environment were used, to increase the set of classifiers tested:

1. sklearn.linear_model.SGDClassifier;
2. sklearn.naive_bayes.GaussianNB;
3. sklearn.naive_bayes.BernoulliNB;
4. sklearn.naive_bayes.MultinomialNB;
5. sklearn.tree.DecisionTreeClassifier;
6. sklearn.ensemble.ExtraTreesClassifier;
7. sklearn.ensemble.RandomForestClassifier;
8. sklearn.ensemble.GradientBoostingClassifier;
9. sklearn.neighbors.KNeighborsClassifier;
10. sklearn.svm.LinearSVC;
11. sklearn.svm.SVC;
12. sklearn.linear_model.LogisticRegression.

After the computational effort using the twelve classifiers, the five best ones were selected, based on the area under the Receiver Operating Characteristics:

1. Linear Model Logistic Regression.
2. Gradient Boosting (Stochastic Gradient Boosting).
3. SVM Linear SVC (Support Vector Machine Linear—Support Vector Classification).
4. Linear Model Stochastic Gradient Descendent (Linear Model SGD).
5. SVM SVC (Support Vector Machine—Support Vector Clustering).

The results of the database simulation are provided in detail in the following sections.

## 5. Results

First-order statistics concern the distribution of gray levels in an image, where the first-order histogram is used as the basis for extracting its characteristics, such as—mean, standard deviation, kurtosis and skewness, as shown in Table 1. These are not sufficient, however, for decision making between what is melanoma tissue and what is healthy tissue. The Mann-Whitney U test applied to the parameters in Table 1 show that (*p* < 0.05), therefore, the null hypothesis is rejected. Meaning that the distributions of both samples (melanoma and nevus) are not the same.

**Figure 4:**
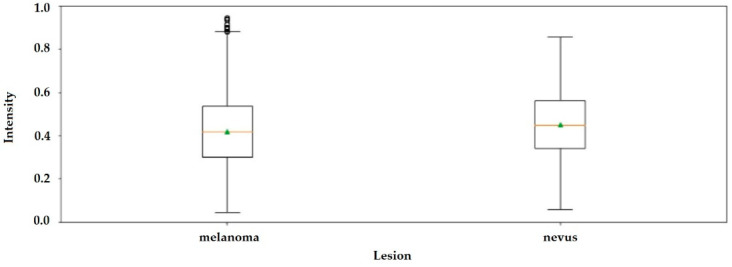
Boxplot with average intensities of grayscale images with melanoma and nevus.

The boxplot in Figure 4 shows that it is not possible to differentiate nevus tissue from tissue with melanoma by only observing the average intensity values.

### Performance Metrics for Classifiers

In academic medical literature, instances are designated as positive, indicating the existence of the disease and negative, indicating the absence of the disease; thus, four possibilities arise when medical images are submitted to the classifiers:

- TP-True Positive: correctly classified positive cases.
- TN-True Negative: correctly classified negative cases.
- FP-False Positive: incorrectly classified negative cases.
- FN-False Negative: incorrectly classified positive cases.

The metrics that were considered to evaluate the classifiers for these were:

1. Accuracy is the ratio of the number of instances correctly classified to the number of all instances in the test suite.

(17) $$Accuracy=\frac{TP+TN}{TP+TN+FP+FN}$$

2. Sensitivity, also known as recall, is the ratio of positives predicted correctly relative to the actual number of positives in the test set.

(18) $$Sensitivity=\frac{TP}{TP+FN}$$

3. Specificity is the version of the sensitivity for negatives and indicates the proportion of negatives correctly predicted relative to the actual number of negatives.

(19) $$Specificity=\frac{TN}{TP+FP}$$

4. The F score is a metric that considers both precision and sensitivity by taking their harmonic mean.

(20) $$F-score=2*\;\frac{recall*precision}{recall+precision}$$

The ideal limit for all metrics is to reach the unit value.

Table 2 shows the five best classifiers in descending order by Area Under the Receiver Operating Characteristics (AUC); these presented the best performance, of the twelve tested.

In this study, considering the test database, the AUC of this set of classifiers, reached levels between 95.04% and 97.46%. All experiments were conducted under the same Python software setup.

Having a unique metric evaluation facilitates the decision-making process for selection of the best classifier from a set of the top five. This metric is the AUC. It provides a clear classification of preferences among all of them and therefore a clear choice of direction. Thus, the best classifier was the Linear Logistic Regression Model.

**Figure 5:**
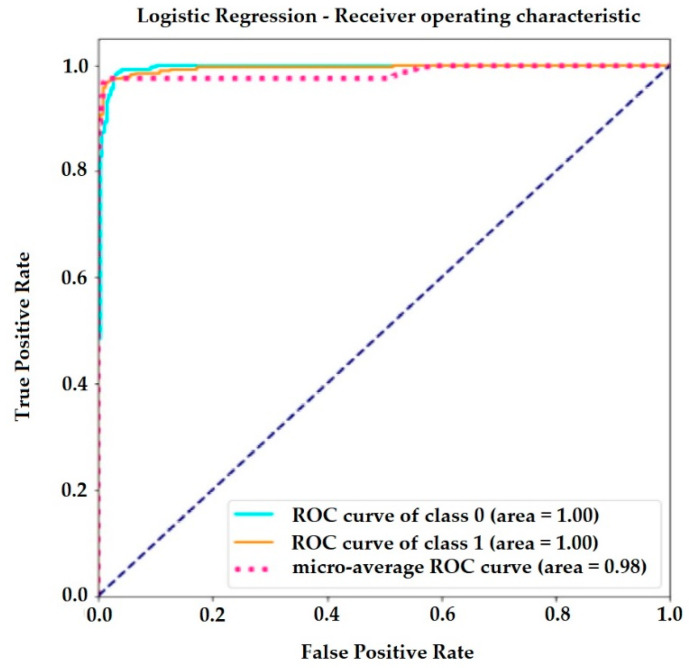
Receiver operating curve (ROC) curve of logistic regression.

The receiver operating curve (ROC) in Figure 5 is another common tool used with the binary classifier. It clarifies resource selection and the accuracy of the logistic regression classifier. The blue dotted line represents the ROC curve of a purely random classifier. A good classifier stays as far away from that line as possible, as here, in the upper left corner.

A suitable overall measure for the curve is the area under the curve (AUC).

**Figure 6:**
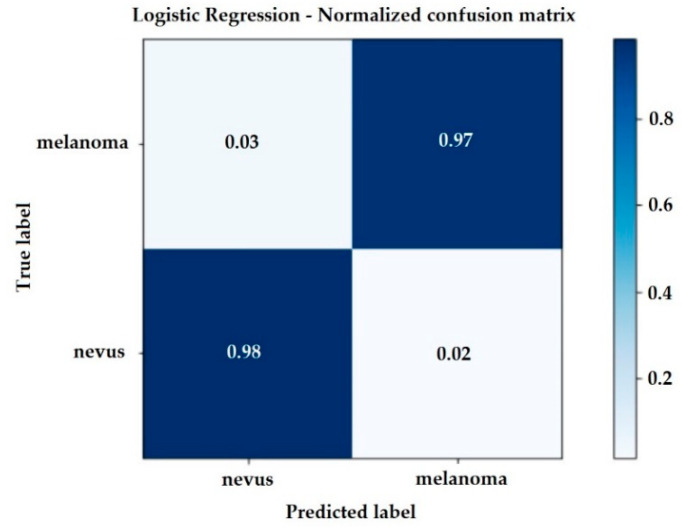
Confusion Matrix considering the Logistic Regression Model.

The confusion matrix of the Logistic Regression method that describes the complete performance of the model is shown in Figure 6. This generates a sensitivity and specificity of 0.97.

The accuracy of a classification can be evaluated by computing the number of correctly recognized class examples (true positives), the number of correctly recognized examples that do not belong to the class (true negatives) and examples that either were incorrectly assigned to the class (false positives) or that were not recognized as class examples (false negatives) [26].

In the Linear Logistic Regression model, the recall for identification of melanoma was 97%. So, of the 500 medical images in the test database, 269 images were of melanoma and the classifier was 97% correct, that is, it identified TP = 261 images correctly, wrongly classifying FN = 8 of the images as nevus.

Similarly, the recall for nevus identification was 98.00%. The classifier recognized TN = 226 images as nevus, classifying FP = 5 images as melanoma, for a total of 231 nevus medical images.

The same reasoning can be made for the other classifiers listed in Table 2.

**Figure 7:**
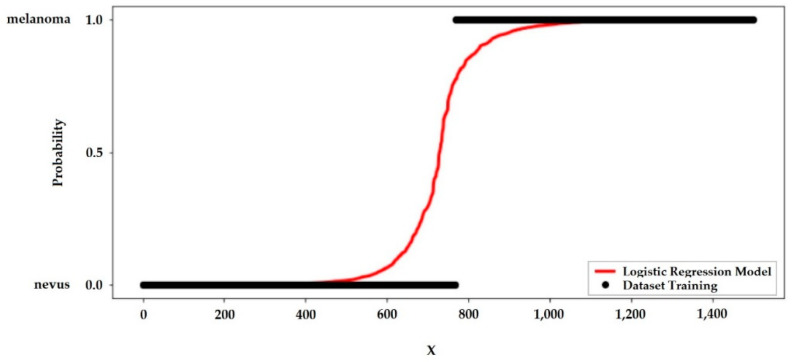
Logistic Regression Model of the Proposed Strategy.

For the best performance model, the probability curve is shown through the sigmoid curve showed in Figure 7.

Just as linear regression uses the least common square method to minimize error and to attain the best possible solution, logistic regression achieves the best results using the maximum likelihood method, plotting the probability curve as a function of the number of samples tested. The steeper this curve, the smaller the sample range that leads to the probability curve 0 < *p* < 1, for diagnosis detection, *p* > 0.5 probably the tissue will be melanoma, if *p* < 0.5 the tissue will be nevus.

A ROC curve is a graphical tool used to understand the performance of a classification model. For a logistic regression model, a prediction can either be positive or negative. Also, this prediction can either be correct or incorrect.

(21) $$False\;Positive\;Rate=\frac{FP}{TN+FP}=1-Specificity.=84.01\%.$$

The Specificity is 15.99%. The number of positive and negative outcomes change as we change the threshold of probability values to classify a probability value as a positive or negative outcome. Thus, the Sensitivity and Specificity will change as well [27].

## 6. Discussion

Although the results were satisfactory with the use of statistical techniques, for future studies, it should be noted that when second order GLCM parameters were used, it is necessary to take some precautions regarding the size of the region of interest.

In some cases, the size of the ROI can change values in some parameters. For example, parameters describing the image homogeneity and complexity (angular second moment, entropy, sum entropy and difference entropy) are examples of parameters that depend on the ROI size, especially with small ROI sizes that approach a limit value [28].

The AUC found in this study, as shown in Table 2, considering the test database, reached levels between 95.04% and 97.46%, which corresponds to an accuracy between 95.00% and 97.00%, respectively. The Linear Model Logistic Regression classifiers were the most accurate.

This shows the effectiveness of second-order statistics and the inclusion of RGB components in the composition of keypoints in improving performance of the proposed strategy.

## 7. Conclusions

The proposed mechanism of identifying and classifying skin tissue is general; in future work it can be applied to other medical images to verify the results, since the strategy analyzes the texture of the images and reveals their differences according to the parameters set, enabling the image classification.

Texture analysis utilizes the changes in the grey value of image pixels and their distribution pattern, which can reflect microscopic pathological changes that are not visible to the human eye and can be used in the analysis of various images. Thus, texture analysis in medical imaging can be a substantial support for the clinical decision-making process in the diagnosis and classification of tumors. This methodology is expected to become more accurate than the human eye in detecting minute deviations in cell and tissue structures.

Statistical methods using GLCM features, associated with red, green and blue color information to perform micro-texture analyzes of human tissues and image classification for tumor detection showed great efficiency in the results presented.

The results show that for the detection of melanoma in human tissues, the logistic regression model was the best model with 97.00% of accuracy and precision on benchmark dataset and also a sensitivity and specificity of 97.00%.

The second-best method of classifying the data of the evaluated medical images was the Classification of the Gradient Boosting.

## Figures and Tables

**Table 1:** First-order statistics.

Parameters	Nevus Tissue	Melanoma Tissue	*p*-Value
Mean Intensity	0.4514 ± 0.14340	0.4204 ± 0.1543	0.000
Kurtosis	3.0953 ± 5.4136	3.6476 ± 6.7590	0.003
Skewness	0.1935 ± 1.7280	0.2479 ± 1.8501	0.022

**Table 2:** Classifiers ordered by Area Under the Receiver Operating Characteristics (AUC).

Classifiers	AUC	Precision	Recall	F1-Score	Support
Logistic Regression	0.9746				
Melanoma		0.98	0.97	0.98	269
Nevus		0.96	0.98	0.97	231
Accuracy				0.97	
Weighted average		0.97	0.98	0.97	500
2. Gradient Boosting	0.9699				
Melanoma		0.97	0.97	0.97	269
Nevus		0.97	0.97	0.97	231
Accuracy				0.97	
Weighted average		0.97	0.97	0.97	500
3. SVM Linear SVC	0.9659				
Melanoma		0.99	0.94	0.97	269
Nevus		0.93	0.99	0.96	231
Accuracy				0.96	
Weighted average		0.96	0.97	0.96	500
4. Linear Model SGD	0.9551				
Melanoma		0.95	0.97	0.96	269
Nevus		0.96	0.94	0.95	231
Accuracy				0.96	
Weighted average		0.96	0.96	0.96	500
5. SVM SVC	0.9504				
Melanoma		0.94	0.97	0.96	269
Nevus		0.96	0.93	0.95	231
Accuracy				0.95	
Weighted average		0.95	0.95	0.95	500

^1^ Linear Model Logistic Regression. ^2^ Stochastic Gradient Boosting. ^3^ Support Vector Machine Linear—Support Vector Classification. ^4^ Linear Model Stochastic Gradient Descendent. ^5^ Support Vector Machine—Support Vector Clustering.

## References

[1] World Cancer Research Fund—American Institute for Cancer Research https://www.wcrf.org/dietandcancer/cancer-trends/skin-cancer-statistics.

[2] Karabulut E.M., Ibrikci T. Texture Analysis of Melanoma Images for Computer-aided Diagnosis. Proceedings of the Annual Int‘l Conference on Intelligent Computing, Computer Science and Information Systems (ICCSIS-16).

[3] Fernandez H.C., Ortega O.L. (2017). Félix Castro-Espinozaa and Volodymyr Ponomaryov. An Intelligent System for the Diagnosis of Skin Cancer on Digital Images taken with Dermoscopy. Acta Polytech. Hung..

[4] Almeida M.A.M. (2018). Use of Statistical Techniques to Analyze Textures in Medical Images for Tumor Detection and Evaluation. Adv. Mol. Imaging Interv. Radiol..

[5] World Health Organization’s (WHO), International Agency for Research on Cancer (IARC) https://www.iarc.fr.

[6] Singh P., Mukundan R., De Ryke R. (2017). Texture Based Quality Analysis of Simulated Synthetic Ultrasound Images Using Local Binary Patterns. J. Imaging.

[7] Zhang L., Gao H.J., Zhang J., Badami B. (2020). Optimization of the Convolutional Neural Networks for Automatic Detection of Skin Cancer. Open Med..

[8] Pathan S., Prabhu G., Siddalingaswamy P. (2018). Techniques and algorithms for computer aided diagnosis of pigmented skin lesions—A review. Biomed. Signal Process. Control..

[9] Lee Y.C., Jung S.H., Won H.H. (2018). WonDerM: Skin Lesion Classification with Fine-tuned Neural Netwporks. ISIC 2018 Lesion Analysis Towards Melanoma Detection.

[10] Harangi B. (2018). Skin lesion classification with ensembles of deep convolutional neural networks. J. Biomed. Inform..

[11] Li Y., Shen L. (2018). Skin Lesion Analysis towards Melanoma Detection Using Deep Learning Network. Sensors.

[12] Abbadi N.K., Faisal Z. (2017). Detection and Analysis of Skin Cancer from Skin Lesions. Int. J. Appl. Eng. Res..

[13] Ansari U.B. (2017). Skin Cancer Detection Using Image Processing. Int. Res. J. Eng. Technol..

[14] Waugh S., Purdie C., Jordan L.B., Vinnicombe S., Lerski R.A., Martin P., Thompson A.M. (2015). Magnetic resonance imaging texture analysis classification of primary breast cancer. Eur. Radiol..

[15] Vamvakas A., Tsougos I., Arikidis N., Kapsalaki E., Fountas K., Fezoulidis I., Costaridou L. (2018). Exploiting morphology and texture of 3D tumor models in DTI for differentiating glioblastoma multiforme from solitary metastasis. Biomed. Signal Process. Control..

[16] Jenitta A., Ravindran R.S. (2017). Image Retrieval Based on Local Mesh Vector Co-occurrence Pattern for Medical Diagnosis from MRI Brain Images. J. Med Syst..

[17] Asri H., Mousannif H., Al Moatassime H., Noel T. (2016). Using Machine Learning Algorithms for Breast Cancer Risk Prediction and Diagnosis. Procedia Comput. Sci..

[18] Yoon S.H., Park C.M., Park S.J., Yoon J.-H., Hahn S., Goo J.M. (2016). Tumor Heterogeneity in Lung Cancer: Assessment with Dynamic Contrast-enhanced MR Imaging. Radiol..

[19] Seal A., Bhattacharjee D., Nasipuri M. (2017). Predictive and probabilistic model for cancer detection using computer tomography images. Multimedia Tools Appl..

[20] Haralick R.M. (1979). Statistical and structural approaches to texture. Proc. IEEE.

[21] Harshavardhan A., Babu S., Venugopal T. (2017). Analysis of Feature Extraction Methods for the Classification of Brain Tumor Detection. Int. J. of Pure Appl. Math..

[22] Bahadure N., Ray A.K., Thethi H.P. (2017). Image Analysis for MRI Based Brain Tumor Detection and Feature Extraction Using Biologically Inspired BWT and SVM. Int. J. Biomed. Imaging.

[23] Abdel-Nasser M., Moreno A., Puig D. (2019). Breast Cancer Detection in Thermal Infrared Images Using Representation Learning and Texture Analysis Methods. Electron..

[24] Ayyachamy S. (2015). Registration Based Retrieval using Texture Measures. Appl. Med Inform..

[25] International Skin Imaging Collaboration https://challenge2019.isic-archive.com/.

[26] Ashish K. (2016). Learning Predictive Analytics with Phyton.

[27] Sokolova M., Lapalme G. (2009). A systematic analysis of performance measures for classification tasks. Inf. Process. Manag..

[28] Sikiö M., Holli-Helenius K.K., Ryymin P., Dastidar P., Eskola H., Harrison L. (2015). The effect of region of interest size on textural parameters. Proceedings of the 2015 9th International Symposium on Image and Signal Processing and Analysis (ISPA).

